# 1-(9-Methyl-11-sulfanyl­idene-8-oxa-10,12-diaza­tricyclo­[7.3.1.0^2,7^]trideca-2,4,6-trien-13-yl)ethanone

**DOI:** 10.1107/S1600536811013699

**Published:** 2011-04-16

**Authors:** Malahat M. Kurbanova, Abel M. Maharramov, Aysel B. Novruzova, Atash V. Gurbanov, Seik Weng Ng

**Affiliations:** a Department of Organic Chemistry, Baku State University, Baku, Azerbaijan; b Department of Chemistry, University of Malaya, 50603 Kuala Lumpur, Malaysia

## Abstract

The six-membered oxacyclo­hexene ring of the title compound, C_13_H_14_N_2_O_2_S, is fused with the benzene ring and the quarternary C atom lies above the plane of the benzene ring by 0.229 (8) Å, whereas the methine C atom (which bears the acetyl substituent) lies below this plane by 0.595 (8) Å. The oxacyclo­hexene ring is also fused with the sofa-shaped 2,6-diaza­cyclo­hexa­none ring. The methine C atom that belongs to both six-membered rings lies above the mean plane of the other five atoms (r.m.s. deviation = 0.077 Å) by 0.759 (5) Å. In the crystal, N—H⋯S hydrogen bonds link adjacent mol­ecules into a linear chain.

## Related literature

For related structures, see: Kettmann & Svetlík (1996 ▶, 1997) ▶; Kurbanova *et al.* (2009 ▶).
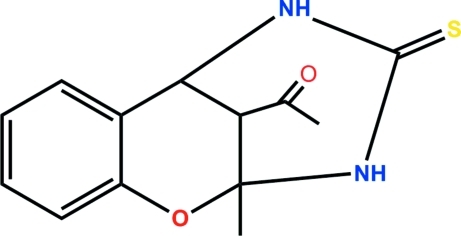


## Experimental

### 

#### Crystal data

C_13_H_14_N_2_O_2_S
*M* *_r_* = 262.32
Monoclinic, 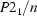

*a* = 8.2382 (5) Å
*b* = 19.1223 (12) Å
*c* = 9.2209 (6) Å
β = 114.623 (1)°
*V* = 1320.51 (14) Å^3^
*Z* = 4
Mo *K*α radiation
μ = 0.24 mm^−1^
*T* = 296 K
0.40 × 0.30 × 0.20 mm

#### Data collection

Bruker APEXII diffractometer
Absorption correction: multi-scan (*SADABS*; Sheldrick, 1996 ▶) *T*_min_ = 0.589, *T*_max_ = 1.000
8830 measured reflections
2292 independent reflections
1523 reflections with *I* > 2σ(*I*)
*R*_int_ = 0.119

#### Refinement

*R*[*F* ^2^ > 2σ(*F* ^2^)] = 0.079
*wR*(*F* ^2^) = 0.211
*S* = 0.99
2292 reflections
164 parameters
H-atom parameters constrained
Δρ_max_ = 0.65 e Å^−3^
Δρ_min_ = −0.41 e Å^−3^

### 

Data collection: *APEX2* (Bruker, 2005 ▶); cell refinement: *SAINT* (Bruker, 2005 ▶); data reduction: *SAINT*; program(s) used to solve structure: *SHELXS97* (Sheldrick, 2008 ▶); program(s) used to refine structure: *SHELXL97* (Sheldrick, 2008 ▶); molecular graphics: *X-SEED* (Barbour, 2001 ▶); software used to prepare material for publication: *publCIF* (Westrip, 2010 ▶).

## Supplementary Material

Crystal structure: contains datablocks global, I. DOI: 10.1107/S1600536811013699/xu5193sup1.cif

Structure factors: contains datablocks I. DOI: 10.1107/S1600536811013699/xu5193Isup2.hkl

Additional supplementary materials:  crystallographic information; 3D view; checkCIF report

## Figures and Tables

**Table 1:** Hydrogen-bond geometry (Å, °)

*D*—H⋯*A*	*D*—H	H⋯*A*	*D*⋯*A*	*D*—H⋯*A*
N1—H1⋯S1^i^	0.88	2.49	3.324 (3)	158
N2—H2⋯S1^ii^	0.88	2.43	3.259 (3)	158

Symmetry codes: (i) 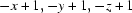; (ii) 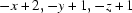.

**Fig. 1.**
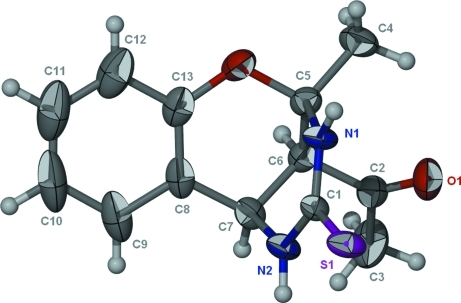
Thermal ellipsoid plot (Barbour, 2001) of the hydrogen-bonded dimeric structure of C13H14N2O2 at the 50% probability level; hydrogen atoms are drawn as spheres of arbitrary radius.

**Fig. 2.**
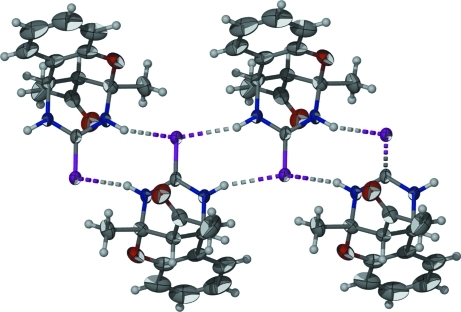
Hydrogen-bonded chain structure.

## supplementary crystallographic information

### Comment

The title compound, C_13_H_14_N_2_O_2_S (Scheme I, Fig. 1), is a conformationally restricted dihydropyrimidine analogue of 1,4-dihydropyridine-type calcium antagonists; the crystal structures of similar compounds have been reported (Kurbanova *et al.*, 2009; Kettmann & Svetlík, 1996; Kettmann & Svetlík, 1997). Tthe six-membered oxacyclohexene ring that is fused with the benzene ring has the quarternary C atom lying above the plane of the benzene ring and the methine C (which bears the acetyl substituent) lying below this plane. The oxacyclohexene ring is also fused with the sofa-shaped diazacyclcohexane ring; the methine C that belongs to both six-membered rings lies above the mean plane of the other five atoms. Hydrogen bonds of the type N–H···S link adjacent molecules to form a linear chain (Fig. 2).

### Experimental

In round-bottom flask that was fitted with a reflux condenser and a mechanical stirrer, salicylaldehyde (1.25 mol), acetylacetone (1.50 mol), thiocarbamide (1.25 mol), trichloroacetic acid (25 mg) and ethanol (10 ml) were reacted for 3 h. The solid that formed was collected and recrystallized from ethanol, m.p. 514–515 K; yield 80%.

### Refinement

Hydrogen atoms were placed in calculated positions [C–H 0.93 to 0.9 and N–H 0.88 7 Å; *U*(H) 1.2 to 1.5*U*(C,N)] and were included in the refinement in the riding model approximation.

### Crystal data

**Table:**

C_13_H_14_N_2_O_2_S	*F*(000) = 552
*M**_r_* = 262.32	*D*_x_ = 1.319 Mg m^−^^3^
Monoclinic, *P*2_1_/*n*	Mo *K*α radiation, λ = 0.71073 Å
Hall symbol: -P 2yn	Cell parameters from 2832 reflections
*a* = 8.2382 (5) Å	θ = 2.7–28.3°
*b* = 19.1223 (12) Å	µ = 0.24 mm^−^^1^
*c* = 9.2209 (6) Å	*T* = 296 K
β = 114.623 (1)°	Irregular block, colorless
*V* = 1320.51 (14) Å^3^	0.40 × 0.30 × 0.20 mm
*Z* = 4	

### Data collection

**Table:**

Bruker APEXII diffractometer	2292 independent reflections
Radiation source: fine-focus sealed tube	1523 reflections with *I* > 2σ(*I*)
graphite	*R*_int_ = 0.119
φ and ω scans	θ_max_ = 25.0°, θ_min_ = 2.1°
Absorption correction: multi-scan (*SADABS*; Sheldrick, 1996)	*h* = −9→9
*T*_min_ = 0.589, *T*_max_ = 1.000	*k* = −22→21
8830 measured reflections	*l* = −10→10

### Refinement

**Table:**

Refinement on *F*^2^	Primary atom site location: structure-invariant direct methods
Least-squares matrix: full	Secondary atom site location: difference Fourier map
*R*[*F*^2^ > 2σ(*F*^2^)] = 0.079	Hydrogen site location: inferred from neighbouring sites
*wR*(*F*^2^) = 0.211	H-atom parameters constrained
*S* = 0.99	*w* = 1/[σ^2^(*F*_o_^2^) + (0.1282*P*)^2^] where *P* = (*F*_o_^2^ + 2*F*_c_^2^)/3
2292 reflections	(Δ/σ)_max_ = 0.001
164 parameters	Δρ_max_ = 0.65 e Å^−^^3^
0 restraints	Δρ_min_ = −0.41 e Å^−^^3^

### Fractional atomic coordinates and isotropic or equivalent isotropic displacement parameters (Å^2^)

**Table:**

	*x*	*y*	*z*	*U*_iso_*/*U*_eq_	
S1	0.70755 (12)	0.51631 (6)	0.41553 (12)	0.0471 (4)	
O1	0.6767 (4)	0.29345 (19)	0.3421 (3)	0.0690 (10)	
O2	0.6966 (4)	0.34703 (14)	0.7965 (3)	0.0491 (8)	
N1	0.6447 (4)	0.40871 (16)	0.5610 (3)	0.0381 (8)	
H1	0.5416	0.4296	0.5371	0.046*	
N2	0.9280 (4)	0.41746 (16)	0.5844 (3)	0.0398 (8)	
H2	1.0075	0.4362	0.5556	0.048*	
C1	0.7642 (4)	0.4431 (2)	0.5272 (4)	0.0358 (9)	
C2	0.8091 (5)	0.2822 (2)	0.4611 (5)	0.0489 (10)	
C3	0.9636 (7)	0.2461 (4)	0.4534 (6)	0.0855 (18)	
H3A	0.9226	0.2139	0.3655	0.128*	
H3B	1.0271	0.2210	0.5510	0.128*	
H3C	1.0417	0.2799	0.4388	0.128*	
C4	0.4941 (5)	0.3002 (2)	0.5599 (5)	0.0584 (12)	
H4A	0.4010	0.3264	0.5719	0.088*	
H4B	0.5078	0.2559	0.6126	0.088*	
H4C	0.4633	0.2927	0.4486	0.088*	
C5	0.6667 (5)	0.3403 (2)	0.6330 (4)	0.0406 (9)	
C6	0.8282 (5)	0.3056 (2)	0.6238 (4)	0.0383 (9)	
H6	0.8597	0.2647	0.6940	0.046*	
C7	0.9783 (5)	0.35889 (19)	0.6938 (4)	0.0405 (9)	
H7	1.0902	0.3386	0.7000	0.049*	
C8	0.9989 (6)	0.3797 (2)	0.8581 (4)	0.0472 (11)	
C9	1.1601 (7)	0.4052 (2)	0.9698 (5)	0.0716 (15)	
H9	1.2577	0.4100	0.9445	0.086*	
C10	1.1735 (10)	0.4238 (3)	1.1220 (6)	0.094 (2)	
H10	1.2805	0.4410	1.1985	0.113*	
C11	1.0297 (11)	0.4164 (3)	1.1571 (6)	0.096 (2)	
H11	1.0396	0.4288	1.2580	0.115*	
C12	0.8736 (8)	0.3917 (2)	1.0492 (5)	0.0747 (16)	
H12	0.7766	0.3869	1.0752	0.090*	
C13	0.8582 (6)	0.3734 (2)	0.8995 (4)	0.0500 (11)	

### Atomic displacement parameters (Å^2^)

**Table:**

	*U*^11^	*U*^22^	*U*^33^	*U*^12^	*U*^13^	*U*^23^
S1	0.0296 (6)	0.0510 (7)	0.0610 (7)	0.0056 (4)	0.0192 (5)	0.0222 (5)
O1	0.0578 (18)	0.099 (3)	0.0400 (16)	−0.0015 (17)	0.0104 (14)	−0.0118 (16)
O2	0.0670 (18)	0.0456 (18)	0.0456 (15)	−0.0074 (14)	0.0341 (13)	−0.0029 (12)
N1	0.0286 (15)	0.0385 (19)	0.0522 (18)	0.0001 (13)	0.0218 (13)	0.0067 (14)
N2	0.0265 (16)	0.0402 (19)	0.0509 (18)	0.0005 (13)	0.0144 (13)	0.0105 (14)
C1	0.0246 (18)	0.045 (2)	0.0375 (19)	−0.0003 (16)	0.0130 (14)	−0.0014 (16)
C2	0.051 (2)	0.053 (3)	0.046 (2)	−0.010 (2)	0.0228 (19)	−0.0091 (19)
C3	0.072 (3)	0.122 (5)	0.063 (3)	0.025 (3)	0.029 (2)	−0.022 (3)
C4	0.053 (3)	0.055 (3)	0.074 (3)	−0.018 (2)	0.033 (2)	−0.010 (2)
C5	0.047 (2)	0.037 (2)	0.043 (2)	−0.0050 (17)	0.0232 (17)	−0.0016 (16)
C6	0.043 (2)	0.035 (2)	0.0365 (18)	0.0000 (16)	0.0171 (15)	0.0004 (16)
C7	0.036 (2)	0.036 (2)	0.044 (2)	0.0025 (16)	0.0102 (15)	0.0059 (16)
C8	0.058 (3)	0.029 (2)	0.039 (2)	−0.0001 (18)	0.0048 (18)	0.0003 (16)
C9	0.076 (3)	0.050 (3)	0.055 (3)	−0.008 (2)	−0.007 (2)	0.000 (2)
C10	0.115 (5)	0.065 (4)	0.050 (3)	−0.010 (4)	−0.016 (3)	−0.011 (3)
C11	0.156 (6)	0.061 (4)	0.047 (3)	−0.012 (4)	0.019 (4)	−0.015 (3)
C12	0.127 (5)	0.045 (3)	0.054 (3)	0.003 (3)	0.038 (3)	−0.005 (2)
C13	0.075 (3)	0.029 (2)	0.042 (2)	−0.001 (2)	0.020 (2)	−0.0014 (17)

### Geometric parameters (Å, °)

**Table:**

S1—C1	1.685 (4)	C4—H4B	0.9600
O1—C2	1.201 (5)	C4—H4C	0.9600
O2—C13	1.369 (5)	C5—C6	1.520 (5)
O2—C5	1.429 (4)	C6—C7	1.523 (5)
N1—C1	1.324 (5)	C6—H6	0.9800
N1—C5	1.443 (5)	C7—C8	1.505 (6)
N1—H1	0.8800	C7—H7	0.9800
N2—C1	1.322 (4)	C8—C13	1.369 (6)
N2—C7	1.447 (5)	C8—C9	1.387 (6)
N2—H2	0.8800	C9—C10	1.407 (9)
C2—C3	1.475 (6)	C9—H9	0.9300
C2—C6	1.509 (5)	C10—C11	1.359 (10)
C3—H3A	0.9600	C10—H10	0.9300
C3—H3B	0.9600	C11—C12	1.342 (8)
C3—H3C	0.9600	C11—H11	0.9300
C4—C5	1.506 (5)	C12—C13	1.377 (6)
C4—H4A	0.9600	C12—H12	0.9300
			
C13—O2—C5	117.1 (3)	C2—C6—C5	117.0 (3)
C1—N1—C5	126.5 (3)	C2—C6—C7	110.5 (3)
C1—N1—H1	116.8	C5—C6—C7	105.0 (3)
C5—N1—H1	116.8	C2—C6—H6	108.0
C1—N2—C7	120.9 (3)	C5—C6—H6	108.0
C1—N2—H2	119.5	C7—C6—H6	108.0
C7—N2—H2	119.5	N2—C7—C8	112.1 (3)
N2—C1—N1	117.2 (3)	N2—C7—C6	106.0 (3)
N2—C1—S1	121.9 (3)	C8—C7—C6	109.5 (3)
N1—C1—S1	120.9 (2)	N2—C7—H7	109.7
O1—C2—C3	120.9 (4)	C8—C7—H7	109.7
O1—C2—C6	122.2 (4)	C6—C7—H7	109.7
C3—C2—C6	116.9 (4)	C13—C8—C9	118.9 (4)
C2—C3—H3A	109.5	C13—C8—C7	120.3 (3)
C2—C3—H3B	109.5	C9—C8—C7	120.8 (5)
H3A—C3—H3B	109.5	C8—C9—C10	118.9 (6)
C2—C3—H3C	109.5	C8—C9—H9	120.6
H3A—C3—H3C	109.5	C10—C9—H9	120.6
H3B—C3—H3C	109.5	C11—C10—C9	119.9 (5)
C5—C4—H4A	109.5	C11—C10—H10	120.1
C5—C4—H4B	109.5	C9—C10—H10	120.1
H4A—C4—H4B	109.5	C12—C11—C10	121.3 (6)
C5—C4—H4C	109.5	C12—C11—H11	119.3
H4A—C4—H4C	109.5	C10—C11—H11	119.3
H4B—C4—H4C	109.5	C11—C12—C13	119.5 (6)
O2—C5—N1	109.7 (3)	C11—C12—H12	120.2
O2—C5—C4	103.5 (3)	C13—C12—H12	120.2
N1—C5—C4	110.0 (3)	C8—C13—O2	122.0 (3)
O2—C5—C6	109.2 (3)	C8—C13—C12	121.5 (4)
N1—C5—C6	108.4 (3)	O2—C13—C12	116.5 (5)
C4—C5—C6	116.0 (3)		
			
C7—N2—C1—N1	−6.7 (5)	C2—C6—C7—N2	60.7 (4)
C7—N2—C1—S1	173.4 (3)	C5—C6—C7—N2	−66.3 (4)
C5—N1—C1—N2	−10.3 (5)	C2—C6—C7—C8	−178.1 (3)
C5—N1—C1—S1	169.6 (3)	C5—C6—C7—C8	54.9 (4)
C13—O2—C5—N1	−71.3 (4)	N2—C7—C8—C13	93.0 (4)
C13—O2—C5—C4	171.3 (3)	C6—C7—C8—C13	−24.3 (5)
C13—O2—C5—C6	47.3 (4)	N2—C7—C8—C9	−87.1 (4)
C1—N1—C5—O2	104.1 (4)	C6—C7—C8—C9	155.5 (4)
C1—N1—C5—C4	−142.7 (4)	C13—C8—C9—C10	0.1 (6)
C1—N1—C5—C6	−15.0 (5)	C7—C8—C9—C10	−179.8 (4)
O1—C2—C6—C5	3.9 (6)	C8—C9—C10—C11	0.0 (8)
C3—C2—C6—C5	−177.7 (4)	C9—C10—C11—C12	0.0 (10)
O1—C2—C6—C7	−116.2 (4)	C10—C11—C12—C13	−0.1 (9)
C3—C2—C6—C7	62.3 (5)	C9—C8—C13—O2	−178.5 (4)
O2—C5—C6—C2	169.3 (3)	C7—C8—C13—O2	1.4 (6)
N1—C5—C6—C2	−71.2 (4)	C9—C8—C13—C12	−0.2 (6)
C4—C5—C6—C2	53.0 (5)	C7—C8—C13—C12	179.7 (4)
O2—C5—C6—C7	−67.8 (3)	C5—O2—C13—C8	−13.2 (5)
N1—C5—C6—C7	51.7 (4)	C5—O2—C13—C12	168.4 (4)
C4—C5—C6—C7	175.9 (3)	C11—C12—C13—C8	0.2 (7)
C1—N2—C7—C8	−73.8 (4)	C11—C12—C13—O2	178.6 (4)
C1—N2—C7—C6	45.6 (4)		

### Hydrogen-bond geometry (Å, °)

**Table:**

*D*—H···*A*	*D*—H	H···*A*	*D*···*A*	*D*—H···*A*
N1—H1···S1^i^	0.88	2.49	3.324 (3)	158
N2—H2···S1^ii^	0.88	2.43	3.259 (3)	158

Symmetry codes: (i) −*x*+1, −*y*+1, −*z*+1; (ii) −*x*+2, −*y*+1, −*z*+1.

## References

[bb1] Barbour, L. J. (2001). *J. Supramol. Chem.* **1**, 189–191.

[bb2] Bruker (2005). *APEX2* and *SAINT* Bruker AXS Inc., Madison, Wisconsin, USA.

[bb3] Kettmann, V. & Svetlík, J. (1996). *Acta Cryst.* C**52**, 1496–1499.

[bb4] Kettmann, V. & Svetlík, J. (1997). *Acta Cryst.* C**53**, 1493–1495.

[bb5] Kurbanova, M. M., Kurbanov, A. V., Askerov, R. K., Allakhverdiev, M. A., Khrustalev, V. N. & Magerramov, A. M. (2009). *J. Struct. Chem.* **50**, 505–509.

[bb6] Sheldrick, G. M. (1996). *SADABS* University of Göttingen, Germany.

[bb7] Sheldrick, G. M. (2008). *Acta Cryst.* A**64**, 112–122. 10.1107/S0108767307043930 18156677

[bb8] Westrip, S. P. (2010). *J. Appl. Cryst.* **43**, 920–925.

